# Effect of community-based intervention on knowledge, attitude, and self-efficacy toward home injuries among Egyptian rural mothers having preschool children

**DOI:** 10.1371/journal.pone.0198964

**Published:** 2018-06-21

**Authors:** Omnia S. El Seifi, Eman M. Mortada, Naglaa M. Abdo

**Affiliations:** 1 Department of Community, Environmental and Occupational Medicine, Faculty of Medicine, Zagazig University, Zagazig city, Arab Republic of Egypt; 2 Health Sciences Department, Health and Rehabilitation Sciences College, Princess Nourah Bint Abdulrahman University, Riyadh city, Kingdom of Saudi Arabia; TNO, NETHERLANDS

## Abstract

**Background:**

Parent’s level of knowledge, state of their attitude, and their self-efficacy are the most incriminated reasons for the faulty application of the first aid measures, particularly in children's home injuries.

**Objectives:**

To assess the effect of a health education intervention on improving knowledge, attitude and self- efficacy of mothers having preschool children about home injuries and the basic first aid measures.

**Methods:**

A pre-posttest evaluation of the effect of a health education intervention on changing knowledge, attitude, and self-efficacy about home injuries and the basic first aid measures of 244 rural Egyptian mothers having preschool children.

**Results:**

About 35% of the male children had home injuries 8 weeks earlier to the study. Mean score of total knowledge increased from 10.21±3.1 in pretest to 18.90 ± 2.6 in posttest, total attitude from 6.19±1.8 to 10.26±2.3 and self-efficacy from 20.75±6.1 to 34.43 ± 10.1 with (p < 0.001) for all changes. Age, education level and previous home injuries were the significant predicting factors for total knowledge, attitude and self- efficacy of the mothers.

**Conclusion:**

Health education improves knowledge, attitude, and self-efficacy of the mothers which were obvious regarding home injuries than first aid measures. There is a need for including knowledge about home injuries in the educational curriculum of high schools and universities and to perform training courses to mothers about first aid measures.

## Introduction

Accidents represent a global community health problem with its consequences of morbidities and mortalities [1, 2]. Accidents could happen in a wide assortment of situations; however, 40% of deaths and half of the injuries occur in and around the home, mainly in the age between one to five years old boys' children [3, 4].

In Egypt, home injuries have turned into a general public health issue and the main cause disabilities in preschool age [5]. However, community development and increase awareness in the Egyptian community had a great obvious effect in minimizing rates of injuries among preschool children, as in the year 1998 it was 72.5%, changed to 50.3% in the year 2003 and 39.8% in the year 2014 [6–8].

Home injuries reflect the character and lifestyle of people, where a new pattern of injury emerges with every new technical or cultural change [9]. Preschool children are vulnerable to many types of home injuries, such as wounds, falls, burns, choking and suffocation, poisoning, and electrical shock. Although these injuries could be prevented, its occurrence may result in death or significant disability [10, 11].

The most challenging duty for mothers is to provide a safe environment for their children to minimize or prevent injury [12]. Prevention of home injuries in children has become an essential objective for children's wellbeing and health promotion [13].

First aid is providing an immediate care for an illness or injury, by a trained but not- specialized person, until getting the specific medical treatment [14]. Improving knowledge, attitude and self-efficacy of the parent's will increase their motivation and self-competence in conducting first aid measures for their children [15]. Therefore; aiming to achieve self-care behavior of caregivers, the objectives of this study were: to assess the effect of a health education intervention on improving the knowledge, attitude and self- efficacy of mothers having preschool children about home injuries and the basic first aid measures.

## Subjects and methods

### Study design, setting, and timing:

Pre-posttest health education intervention was conducted over 5 months (from June to the end of October) 2016, in "El Ghar village- Zagazig district-Sharkia governorate", which was randomly selected by a simple random sampling technique out of 74 rural villages in Zagazig district. El Ghar village is a small village, with population size about 10152, including 5944 male and 4208 female [16].

### Study population

The subjects recruited for this study were only; mothers having at least one preschool child within the age from 1 till 5 years old, literate and willing to participate in this study. Those who didn't have any preschool child, or have any mental disorder, or refused to participate were excluded from this study sample.

### Sample size and sampling technique

The sample size was computed using Epi info 7 program depending on; 95% confidence limit, power of 80%, effect size represented in the mean difference of self-efficacy from pre to post test 2.3 as reported from a similar previous study [17], resulting in a sample of 170 mother, the number was duplicated due to the usage of cluster sampling technique leading to total sample size of 340. Ninety six mothers refused to participate in our study, giving a response rate of 71.7% and resulting in a total sample size of 244 mothers who were included in the study and undergo intervention (pretest, health education, and posttest).

Cluster random sample was used for choosing the mothers, "El Ghar village" has been divided into 5 squares depending on boundaries determined during polio campaigns, from which 3 squares were chosen randomly, then systematically every 5^th^ house within the streets was visited, and all mothers who met the inclusion criteria were invited to join this research.

### Intervention procedures

#### A) Tools of data collection

A Structured anonymous self-administrated pre-posttest questionnaire was developed to assess knowledge, attitude, and self-efficacy of the mothers toward the common home injuries among preschool children (poisoning by drugs and chemicals, burns, wound/ facture and chocking), it was divided into different parts:

- General characteristics of the participants: including; mother’s age, education, occupation, family size, the number of children and their genders, the occurrence and the type of home injury if any, 8 weeks earlier to the study.
- Knowledge of the mothers: about the predisposing factors, ways of prevention of the selected types of injuries and the immediate measures that should be taken, were tested by 25 questions; 4 questions were related the general knowledge about home injuries, 5 questions were related to drug & chemical poisoning, 5 question for burns, 6 for wound/ fracture and 5 for choking. Each question has to be answered either by Yes, No or I don't know. The answer with Yes was scored as "1", while both answers with No or I don't know were scored as "0", resulting in a range of knowledge ranging from 0–25. At the end of the knowledge section, they were asked to assign the source of their knowledge.
- Mother’s attitude: in case of exposure to home injuries, this section includes 11 question, the response was measured with a modified 3 point Likert scale (0: disagree, 1: neutral and 2: agree), every mother has the chance to gain a score ranging from 0 to 22.
  The above parts of the questionnaire designed by the researchers based on questionnaires used in previous studies [18, 19].They were validated by the opinion of different independent professional expertise and tested for reliability where Cronbach’s α coefficient equal 0.82 & 0.85 for knowledge and attitude respectively.
- Mother's self-efficacy: mothers were tested by 11 questions about self-caring for their children, these questions were designed based on the validated Sherer’s General Self-efficiency Standard Scale (SGSES) [20], available at< http://dx.doi.org/10.2466/pr0.1982.51.2.663>, which was primarily designed to assess the expectations of the individuals when facing new and stressful situations, the questions of the original scale were modified to be suitable to the problem under study. The response to the questions consiste of a 5-point scale ranging from (0 = strongly disagree to 4 = strongly agree), taking in consideration presence of 5 negative statements with a revised score. Summation of the scores is reflecting the general self-efficacy, ranged from 0 to 44. The higher the total score the more self-efficacy of the participant.
  A pilot study was conducted to evaluate the questionnaire before starting the practical phase of the study. It was carried out on 24 mothers who were excluded from the final sample and statistical analysis. Necessary modifications in the questionnaire were conducted accordingly to make it simpler, shorter and clearer.

#### B) Intervention

The practical phase of our study includes:

- ***Pre-intervention phase*:** The duration of this phase is one month, started by the first of June till the end of the same month, 2016. Home visits were conducted by the researchers and data about baseline knowledge, attitude and self-efficacy were pretested by interviewing every mother individually. The average number was 6–8 mothers per day for about 15–20 minutes depending on the response of each mother.
- ***Intervention phase*:** All participated mothers were undergoing health education intervention over one month from the first of July till the first of August, 2016. Every 8–10 mothers live in the same house and/or nearby houses were grouped and the intervention was delivered to them over 45 minutes, using a scientific prepared message depending on the guidelines available from Centers for Disease Control and Prevention (CDC) about child safety and injury prevention [21] and from a previous similar study [22]. The message was delivered using question and answer approach to ensure the involvement of all mothers. Our message focused on factors related to the occurrence of different types of injuries, immediate required actions and methods of prevention particularly the simple inexpensive ways that make the home safer for children. We didn't miss the importance of increasing Mother's increase self-efficacy through persuasion and role models. These have been vigorously demonstrated & supported by a powerpoint presentation and 2 educational short videos displayed on a laptop, followed by group discussion with the mothers about the contents. Also, pamphlets containing attractive images and clear simple texts were distributed to these mothers to be their guideline after the intervention.
- ***Post-intervention phase*:** This phase was conducted over one month, started by the first of October till the end of October 2016. The change in knowledge, attitude, and self-efficacy of the all included mothers in our study were post-tested after 2 months from health education intervention.

### Measured outcomes

Knowledge, attitude and self- efficacy of mothers about home injuries and immediate first aid measures for injured children.

### Ethical consideration

“All procedures performed in studies involving human participants were in accordance with the ethical standards of the institutional and/or national research committee and with the 1964 Helsinki declaration and its later amendments or comparable ethical standards.” Official approval by ZU-IRB was obtained with a number 4336/4-2-2016, and a written informed consent from mothers was taken after full explanation of the study, it included personal data about the participants and details of the study (title, objectives, expected benefits and risks and confidentiality of data).

### Data management and analysis

The data were recorded, represented and analyzed using SPSS (Statistical Package for the Social Sciences) version 19.0 [23]. Quantitative and qualitative data were presented and summarized using mean, standard deviation (SD) and percentage respectively. Comparing the mean difference before and after the intervention was performed using a paired t-test, the percentage of total knowledge, attitude and self-efficacy were calculated by dividing actual score on the maximum score and multiply by 100. Finally, linear regression analysis was conducted to identify the factors predicting post-intervention knowledge, attitude, and self-efficacy. Statistical significance was considered at p-value ≤0.05.

## Results

Table 1 describes the general characteristics of the study participants; where their mean age was 29.8 ± 5.4, most of them have preparatory education level (37.7%), housewives (81.1%), with family size ≤ 4 members (36.9%). The most common source of mother's knowledge about home injuries was mass media/the internet (32.4%).

**Table 1:** General characters of the studied mothers.

Variables	No (244)	%(100.0)
**Mother's Age (years)**		
mean± S.D	29.8 ± 5.4	
Range	19–38	
**Educational level:**		
Primary education	41	16.8
Preparatory education	92	37.7
Secondary education	62	25.4
University and above	49	20.1
**Occupation:**		
Housewives	198	81. 1
Working	46	18.9
**Family size:**		
≤ 4 members	90	36.9
Five members	68	27.9
Six members	42	17.2
≥ 7 members	44	18.0
**Knowledge sources about home injuries:**		
Mass media	79	32.4
Relatives	67	27.5
Physicians	50	20.5
Campaigns	45	18.4
Others	3	1.2

The participated mothers were having 672 children aged (1–5 years), where 34.8% of them exposed to home injuries in the last 8 weeks before the study, 56.4% of the injured children were males 60.3%, in the age from 1–3 years old. The most reported type of home injury was wounds 41.0%, while the least one was choking 8.6%. Table 2.

**Table 2:** Occurrence and types home injuries among the preschool children.

Items	No. of preschool children of participated mothers (672)	%(100.0)
**The occurrence of home injuries:**		
Not Occurred	438	65.2
Occurred	234	34.8
	**No.(234)**	**% (100.0)**
**-Child's age:**		
1–3 years	132	56.4
3–5 years	102	43.6
**-Child's sex:**		
Male	141	60.3
Female	93	39.7
**Types of home injuries among the affected children:**		
Wounds	96	41.0
Fall/fracture	53	22.6
Poisoning	40	17.1
Burn	25	10.7
Choking	20	8.6

Regarding mother's knowledge, attitude and self-efficacy about prevention of home- injuries and basic first aid measures, Table 3 presenting; a significant increase in the mean score of all studied outcomes in the post-intervention than pre-intervention (p < 0.001) except for attitude toward immediate first aid measures (p = 0.078). For knowledge; the score of change from pre to post intervention was higher regarding wound/fracture (1.95 ± 0.7) and least regarding burn (1.22 ± 0.3), for attitude the score of change regarding prevention of home injuries was higher than that for immediate first aid measures (3.99 ± 1.1 and 0.08 ± 0.3) respectively. Percent of change in total knowledge was 34.8%, total attitude (18.5%) and self-efficacy (31.1%).

**Table 3:** Mother's knowledge, attitude, self-efficacy about prevention of home injuries and basic first aid measures.

Items	Pre-intervention X±SD	Post-intervention X±SD	Score of change	^a^*p
**Knowledge**				
General knowledge about home injuries	2.02 ± 0.4	3.94 ± 0.7	1.92 ± 0.9	0.000
poisoning by drugs and chemicals	2.13 ± 0.6	3.88 ± 0.7	1.74 ± 0.8	0.000
wound/ fracture	2.32 ± 0.5	4.27 ± 0.8	1.95 ± 0.7	0.000
burn	2.09 ± 0.4	3.32 ± 0.8	1.22 ± 0.3	0.000
choking	1.63 ± 0.4	3.48 ± 0.9	1.85 ± 0.5	0.000
**Total Knowledge Score**	**10.21 ± 3.1**	**18.90 ± 2.6**	**8.69 ± 2.6**	**0.000**
**Total Knowledge Percent**	**40.8%**	**75.6%**	**34.8%**	
**Attitude**				
Attitude toward prevention of home injuries	4.05 ± 1.1	8.05 ± 1.5	3.99 ± 1.1	0.000
Attitude toward immediate first aid measures	2.13 ± 0.5	2.21 ± 0.5	0.08 ± 0.3	0.078
**Total Attitude Score**	**6.19 ± 1.8**	**10.26 ± 2.3**	**4.07 ± 1.6**	**0.000**
**Total Attitude Percent**	**28.1%**	**46.6%**	**18.5%**	
**Self- efficacy Score**	**20.75 ± 6.1**	**34.43 ± 10.1**	**13.68 ± 3.8**	**0.000**
**Self- efficacy Percen**t	**47.1%**	**78.2%**	**31.1%**	

*p < o.ooo1 is significant

^a^ Paired t-test was computed.

**Fig 1:**
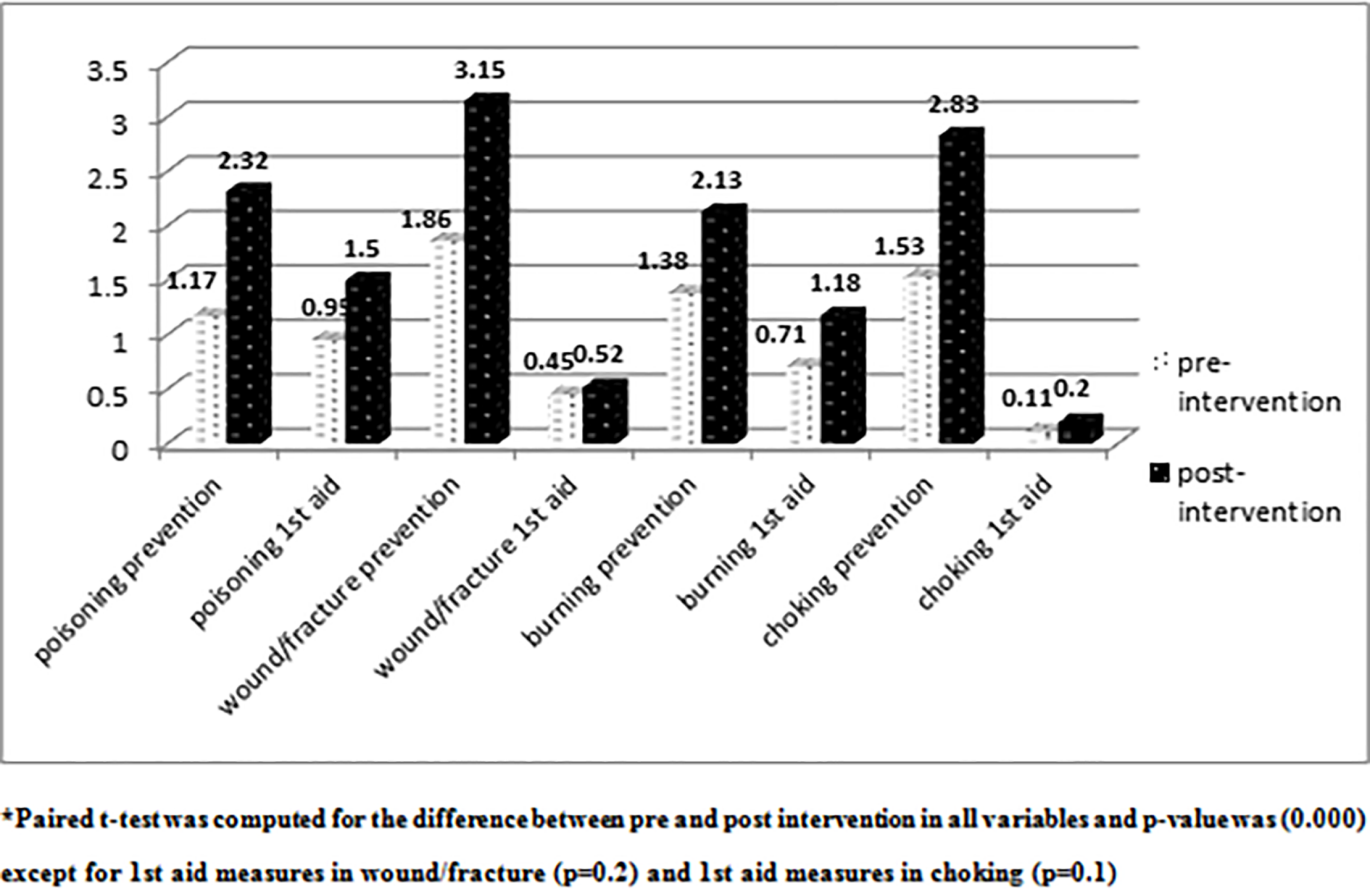
Mean score of knowledge of the participated mothers regarding prevention and first aid measures to the four types of home injuries under study.

Fig 1 demonstrates the mean score of knowledge of the participated mothers regarding prevention and first aid measures to the four types of home injuries under study, where highest mean score of prevention was for wound/fracture in both pre and post intervention (1.86 &3.15) respectively, while lowest mean score was for poisoning prevention in pre-intervention (1.17) and burn prevention in post-intervention (2.13). For first aid measures highest mean score was for poisoning in both pre and post-intervention (0.99 & 1.5) respectively, while the lowest one was for choking in pre-intervention (0.11) and post-intervention (0.20), with statistically significant difference in all variables between pre-intervention and post-intervention (p = 0.00) except for first aid measures in wound/fracture (p = 0.2) and 1^st^ aid measures in choking (p = 0.1).

Age, educational level and previous occurrence of home injuries were the significant predicting factors for post-intervention total knowledge, attitude, and self- efficacy regarding domestic accidents Table 4.

**Table 4:** Regression analyses of factors significantly predict post-intervention total knowledge, attitude, and self-efficacy for mothers about home injuries.

Variable	B	SE	t	*P value	95.0% CI interval for B	
					Lower bound	Upper bound
**Knowledge**						
Age	0.200	0.056	3.575	0.00	0.090	0.309
**Attitude**						
Education	0.769	0.177	4.357	0.00	0.421	1.117
**Self-efficacy**						
Occurrence of home injuries	2.433	0.967	2.515	0.013	0.527	4.338

*p < o.o5 is significant

## Discussion

Parents especially mothers have an important role in an important role in providing a safe home environment for their children in order to minimize or prevent home injuries. Our study was conducted among 244 rural mothers having preschool children to assess the effect of health education intervention on their knowledge, attitude, and self- efficacy about home injuries and the basic first aid measures.

Our study revealed that (34.8%) from the preschool children showed home injuries 8 weeks earlier to the study; this is quite similar to what was found in two different studies were conducted in Egypt (39.8% & 38.8%) [8, 24], as well as to a study conducted in Turkey (36.5%) [25], while it was higher than that reported in India (23%) [26]. This variation could be attributed to that the later study include children up to 14 years old " this age is having a better developmental coordination and more obedient to the orders", also the history of accidents was reported over a year earlier to the study, this long time may lead to under-reporting of home-related injuries.

Among the injured children, the age from 1–3 years was the most affected (56.4%), which is consistent with other similar studies in Egypt and Turkey [8, 24, 25], as children at that age start to move freely at home, explore their surroundings, but they are still physically and mentally immature and unable to distinguish risk, that is why they are exposed to various types of accidents. Male children were recorded to be the most affected by injuries (60.3%), the same result has been shown in other studies that have been conducted in Egypt, Turkey, and India [8, 23, 25], this may be due to the more active and rushing behavior of boys than girls.

Most of our children were exposed to: wounds fall/fracture, drug or chemical poisoning, burn and chocking. This could be contributed to the small size of houses in rural areas where the safety measures are too difficult to be applied; also there is no separate place for children to play in. These types of injuries were to a greater extent the same as reported by other studies conducted in Egypt [24]. Although the developed countries usually have different forms of health problems than the developing one, a study conducted in the USA shows that; falls, burns, poisonings, choking/suffocations were the most common types of children's home injuries [27].

The significant improvements in the general knowledge of the mother about home injuries and its subtypes were consistent with the impact of other intervention studies, where the health education leads to a positive improvement of mother's knowledge about home injuries [17,28].

Although the highest mean score of the change in the knowledge was for wound/fracture, the score of knowledge about basic first aid was low, which is in line with the results of a study that was conducted in India [29]. This may be because acquiring knowledge about ways of prevention is much easier than that for first aid measures, the later one is a more complicated issue that needs repeated training and health education sessions to gain effect. Also, in rural areas some traditional methods are still used for treating injuries till now like putting the coffee on the wounds to stop bleeding, that matter was documented from the results of a study conducted in Upper Egypt [30].

There was a minimal role of physicians and health campaigns as sources of mothers' knowledge compared to mass media and relatives. This could be attributed to the fact that culture in rural areas maximizes the role of relatives' experience, over the role of the healthcare members. This is the same as the results of a study conducted in India; where media, parents and family members were the main source of knowledge [29].

The attitude of the mothers regarding prevention of home injuries was significantly higher in posttest, in our opinion this has been achieved mainly by changing the faulty beliefe of mothers that accidents occurred by chance. However, the improvement in posttest toward first aid measures was non- significant, this brings us to the same conclusion that first aid needs more time to be well established.

Mother's self- efficacy, which is the mother's belief that they can successfully carry out the desired behavior [31], was significantly improved after the intervention, which is consistent with the studies conducted in Iran, concluded that; health education can improve self-efficacy of mothers [32, 33].

From our point of view, this is one of the important achievements of our study as improving self-efficacy will help the mothers to overcome any surprising and difficult situation in caring of their children, which in turn will help in injuries prevention and providing immediate first aid measures.

Our health education message was supported using multimedia aids "pamphlet and video films", which result in higher percentage of change of total knowledge from pre to posttest by (34.8%), followed by self-efficacy (31.1%) then total attitude (18.5%), this is supported by study in Canada which found that using film videos in the education of the mothers about home injuries had positive effect on mother's knowledge, attitude, and involvement [34]. In our opinion, these changes are important and promising for changing future behaviors of the studied mothers, resembling that study which was conducted in Iran, it shows that knowledge and self-efficacy were the most important predictors of maternal behavior in the prevention of home injuries in preschool children [35].

Age of the mothers was the main factor that significantly predicts their total knowledge score, may be related to increasing experience and awareness with age. This result is consistent with the study performed in India, which stated that mothers with age group from 30–40 years old were having a higher knowledge than others [29].

The total attitude score was significantly affected by education of the mothers, which is in the same line with the results of the study conducted in Egypt, demonstrated that the highly educated mothers have better attitude and practice [8].

The previous occurrence of home injuries was significantly affecting self-efficacy of the mothers, this could be due to the emotional, financial and social consequences that follow the occurrence of injuries, which may drive and empower the mothers to be more confident in taking protective actions for their children. This is consistent with the results of a study performed in India, where the previous occurrence of accidents affects the outcomes of health education intervention [29].

### Limitation of the study

There is no specific scale to measure the self-efficacy of mothers in case of injuries, so we were forced to use the general one, which was not suitable for some items our research. Also, the self-reported data may lead to some sort of inaccuracy, as well as lack of enough time to assess the impact of this intervention on the incidence of home injuries.

## Conclusion

Application of health education intervention among mothers having preschool children improves their knowledge, self-efficacy, and attitude about home injuries; however, the improvement in first aid measures applied after injuries is much lower than that for accidents prevention. Age of the mothers, education level and previous occurrence of home injuries were the significant predicting factors for knowledge, attitude, and self-efficacy of the mothers respectively. Therefore our recommendations are:

1. Several follow-up studies to determine the impact of a health education intervention on decreasing rate of home injuries.
2. The need for including knowledge about prevention of home injuries in the educational curriculum of high schools and University.
3. Training courses about first aid measures for the mothers to develop community-based awareness and sound practice.

## Supporting information

S1 Fig Official ethical approval by ZU-IRB. (TIFF)

S1 Appendix Questionnaire, English version. (DOCX)

S2 Appendix Questionnaire, Arabic version. (DOCX)
