# Supplementary figures and images for: Effect of community-based intervention on knowledge, attitude, and self-efficacy toward home injuries among Egyptian rural mothers having preschool children
Source: PLoS One. 2018 Jun 21;13(6):e0198964. doi: 10.1371/journal.pone.0198964 (PMC6013117; doi:10.1371/journal.pone.0198964)

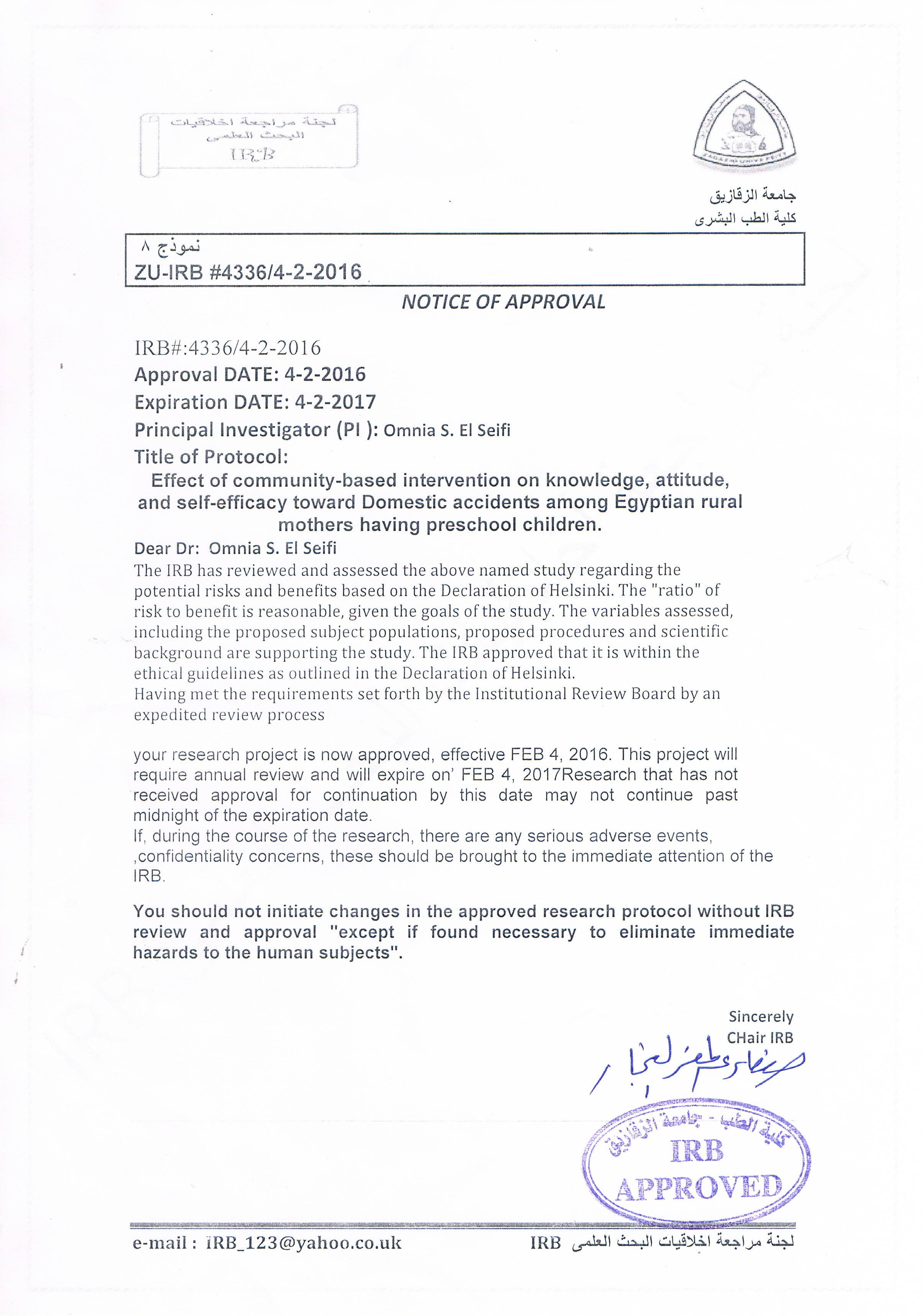

Supplement: S1 Fig — (TIFF) [file pone.0198964.s001.tiff]
