# Supplementary material for: Effect of community-based intervention on knowledge, attitude, and self-efficacy toward home injuries among Egyptian rural mothers having preschool children
Source: PLoS One. 2018 Jun 21;13(6):e0198964. doi: 10.1371/journal.pone.0198964 (PMC6013117; doi:10.1371/journal.pone.0198964)
Supplement: S1 Appendix — (DOCX) [file pone.0198964.s002.docx]

**Pre- post test questionnaire**

**to assess**

**Effect of community-based intervention on knowledge, attitude, and self-efficacy toward home injuries among Egyptian rural mothers having preschool children.**

We are researchers from faculty of medicine, Zagazig University, conducting this research to assess the effect of health education on knowledge, attitude and self-efficacy of mothers toward home injuries in children aged (1-5 years old), we hope your cooperation in filling this questionnaire.

**General characters:**

**Mother's Age:**

**Educational level: ( )** Primary education ( ) Preparatory education ( ) Secondary education ( ) University and above.

**Occupation: ( )** Housewives **( )** Working**.**

**Family size: ( )** ≤ 4 members **( )** Five members **( )** Six members **( )** ≥ 7 members.

**Number of children between (1-5) years old:**

**Child's age: ( )** 1-3 years **( )** 3-5 years**.**

**Child's sex:** ( ) Male ( ) Female.

**Have your children suffered an injury in the past 2 months?** ( ) yes ( ) no

If the answer is yes:

Please mention the type of injury.....

**Knowledge:**

Please choose one answer for each question.

| **Knowledge** | **Yes** | **No** | **I don't know** |
| --- | --- | --- | --- |
| 1. Home injuries are an important cause of injury and death for pre-school children. |  |  |  |
| 1. Home injuries can be prevented by following safety conditions in the home |  |  |  |
| 1. Do you have any information about the first aid that should be followed in case of home injuries? |  |  |  |
| 1. Should the mother not leave the young child to play alone? |  |  |  |
| 1. Leaving detergents and medicines at the bottom of the cabinet is considered dangerous for children. |  |  |  |
| 1. It's important to put locks on the cabinets used to store the drugs and detergents |  |  |  |
| 1. Is the right place to keep the detergent and medicines the highest storage of the cabinet |  |  |  |
| 1. If a child swallows any chemicals, should you make him vomit in all cases? |  |  |  |
| 1. The child should drink milk and egg when he swallows any chemicals |  |  |  |
| 1. leaving sharp instruments in front of the child may expose him to the risk of injuries |  |  |  |
| 1. In the event of a child's injury, the first action to be performed is to pressure the wound. |  |  |  |
| 1. In your opinion, is it correct to leave the child to go up and down the stairs alone without help? |  |  |  |
| 1. Mother does have to choose the right games for the baby to avoid the pointed ones. |  |  |  |
| 1. Do you think, it's correct not to move the child's limb, if you suspect the occurrence of fracture? |  |  |  |
| 1. Should you prevent your child from walking and running over the wet floors? |  |  |  |
| 1. Should restrictions be placed on children entering the kitchen? |  |  |  |
| 1. When hot oil or boiled water falls on a child's chest, will the first step be to remove his clothes? |  |  |  |
| 1. In case of burn, is it correct to put ice packs on the affected part as the first action? |  |  |  |
| 1. Hot food and boiled water should be removed from the front eyes of the cooker |  |  |  |
| 1. Do you have to test the water temperature before giving the child a shower? |  |  |  |
| 1. In case of choking, the first thing to do is to make the child's head lower and hitting down on his back. |  |  |  |
| 1. Removal of small stuffs from the front the child may reduce risk of choking? |  |  |  |
| 1. We should put small and suitable amounts of the food in the child's mouth? |  |  |  |
| 1. Is it necessary to make sure that the baby chews the food well before swallowing? |  |  |  |
| 1. - Should the child be prevented from laughing while eating? |  |  |  |

**What is/ are the sources of your Knowledge about home injuries? (More than one answer is allowed)**

( ) Mass media ( ) Relatives ( ) Physicians ( ) Campaigns ( ) others.

**Attitude:**

Please choose one answer for each question.

| **Attitude** | **Agree** | **Some degree** | **Don't agree** |
| --- | --- | --- | --- |
| 1. Do you support including the basics of first aid and how to avoid injuries in the curriculum of schools and universities |  |  |  |
| 1. Do you think it is important to have a full first aid bag in your home? |  |  |  |
| 1. Do you think that raising awareness of families about home injuries will reduce their incidence? |  |  |  |
| 1. How would you rate this statement “home injury is preventable”? |  |  |  |
| 1. Do you want to attend awareness seminars on how to deal and avoid home injuries? |  |  |  |
| 1. In the event of any of the home injuries, do you think that the first action to be taken is to request health service at the nearest hospital or health unit? |  |  |  |
| 1. Do you think that the use of traditional methods in the treatment of home injuries may be useful in treating them? |  |  |  |
| 1. When any of the home injuries occur, do you think the first thing to do is to consult with relatives or neighbors? |  |  |  |
| 1. Do you think it is important to monitor the mother of her child all the time and not leave him alone at home? |  |  |  |
| 1. Do you think you are able to do your child's first aid in the event of any injury before going to the hospital? |  |  |  |
| 1. Do you think it is easy to protect your child from exposure to home injuries? |  |  |  |

**Self-efficacy:**

Please choose one answer for each question.

| **Self- efficacy** | **Strongly agree** | **Agree** | **Some times** | **Disagree** | **Strongly disagree** |
| --- | --- | --- | --- | --- | --- |
| 1. I am able to apply first aid to my child in emergency situations before seeking for health care |  |  |  |  |  |
| 1. I am able to educate my family on first aid for various injuries |  |  |  |  |  |
| 1. If I plan to prevent home accidents, I am able to do so. |  |  |  |  |  |
| 1. If I fail to prevent home injuries once, I will not give up and I will still try to make it even more successful |  |  |  |  |  |
| 1. If I fail to prevent home injuries once, I will not try again |  |  |  |  |  |
| 1. I will immediately implement the safety measures at my home |  |  |  |  |  |
| 1. The implementation of measures to prevent home injuries is difficult for me so I cannot do it |  |  |  |  |  |
| 1. Failure just makes me try harder |  |  |  |  |  |
| 1. I feel insecure about my ability protect my child |  |  |  |  |  |
| 1. I give up easily. |  |  |  |  |  |
| 1. I do not seem capable of dealing with household injury prevention measures |  |  |  |  |  |
