# Supplementary material for: Effect of community-based intervention on knowledge, attitude, and self-efficacy toward home injuries among Egyptian rural mothers having preschool children
Source: PLoS One. 2018 Jun 21;13(6):e0198964. doi: 10.1371/journal.pone.0198964 (PMC6013117; doi:10.1371/journal.pone.0198964)
Supplement: S2 Appendix — (DOCX) [file pone.0198964.s003.docx]

**استبيان عن المعلومات والمواقف والفاعلية الذاتية للأمهات**

**تجاه الحوادث المنزلية**

**مجموعه من الباحثين بكلية الطب البشرى – جامعة الزقازيق بصدد القيام ببحث الغرض منه معرفة تأثير برنامج التثقيف الصحى على المعلومات والسلوكيات والفاعليه الذاتية تجاه الحوادث المنزلية للاطفال من عمر (1-5) سنوات... نرجو تفاعلكم مشكورين فى الاجابه على الاستبيان**

**معلومات عامة:**

- **عمر الام:**

**- التعليم: ( )** تقرأ وتكتب ( ) تعليم متوسط ( ) تعليم ثانوى ( ) جامعى فما فوق

**- الوظيفة** : ( ) ربة منزل ( ) تعمل.

**- عدد افراد الاسرة**: ( )اقل من او يساوى 4 ( ) 5 افراد ( ) 6 افراد اكثرمن او يساوى 7 افراد.**( )**

**- عدد الاطفال فى عمر مابين (1-5 سنوات):**

**العمر:** ( ) من1- 3 سنوات ( ) من 3-5 سنوات.**-**

**- النوع:** ( ) ذكر ( ) انثى **( يذكر العدد)**

**هل تعرض طفلك للاصابة المنزليه خلال الشهرين السابقين؟؟ ( )** نعم ( ) لا**-**

**اذا كانت الاجابة بنعم:** اذكرى نوع الاصابة**..........**

**المعلومات**

**برجاء اختيار اجابة واحده لكل سؤال**

| **المعلومات** | **نعم** | **لا** | **لا أعلم** |
| --- | --- | --- | --- |
| 1-تعتبر الحوداث المنزليه سببا هاما من اسباب الاصابات والوفيات للاطفال قبل سن المدرسة |  |  |  |
| 2-الحوادث المنزليه من الممكن منع حدوثها باتباع شروط السلامة داخل المنازل |  |  |  |
| 3- هل لديك اى معلومات عن الاسعافات الاوليه التى يجب اتباعها فى حالة حدوث اصابات منزليه؟ |  |  |  |
| 4 هل يجب على الام عدم ترك الطفل الصغير للعب منفردا ؟ |  |  |  |
| 5- ترك المنظفات والادوية اسفل الجزانة يشكل خطر بالنسبة للاطفال ؟ |  |  |  |
| 6- من الهام وجود اقفال على الخزائن المستخدمة فى حفظ الادوية والمنظفات |  |  |  |
| 7- هل المكان الصحيح للاحتفاظ بالادوية والمنظفات اعلى الخزانة؟ |  |  |  |
| 8- فى حالة بلع الطفل لاى مادة كيماويه هل يجب جعله يتقيا فى جميع الحالات؟ |  |  |  |
| 9- فى حالة بلع الطفل لاى مادة كيماوية هل يجب جعله يشرب بيض ولبن؟ |  |  |  |
| 10- ترك الادوات الحادة امام الطفل قد تعرضه لخطر حدوث جروح؟ |  |  |  |
| 11- في حالة حدوث جروح للطفل، هل أول تصرف يجب القيام يه هو بالضغط على الجرح؟ |  |  |  |
| 12- برأيك هل من الصحيح ترك الطفل لصعود ونزول الدرج وحده بدون مساعده؟ |  |  |  |
| 13- برايك هل على الام اختيار الالعاب للطفل بحيث تكون غير مدببه؟ |  |  |  |
| 14- فى رايك هل التصرف الصحيح فى حالة وقع الطفل والشك بوجود كسر هو عدم تحريك الجزء المصاب؟ |  |  |  |
| 15- هل يجب منع الاطفال من السير والجرى على ارضيات الشقه بعد مسحها بالمياه |  |  |  |
| 16-هل يجب وضع قيود على دخول الاطفال للمطبخ؟ |  |  |  |
| 17- عند وقوع الزيت الساخن او المياة المغلية على صدر طفل هل تكون أول خطوة هي إزالة ملابس الطفل؟ |  |  |  |
| 18- في حالة حدوث حرق هل من الصحيح بوضع كمادات ثلج على الجزء المصاب كأول تصرف؟ |  |  |  |
| -19يجب ابعاد الطعام الساخن والماء المغلى على العيون الامامية للبوتاجاز |  |  |  |
| -20 هل يجب اختبار درجة حرارة المياة قبل اعطاء دش للطفل الصغير ؟ |  |  |  |
| 21- فى حال حدوث شرقه للطفل فهل اول ما يجب عمله هو جعل راس الطفل للاسف والخبط باتجاه الاسفل على اعلى ظهره؟ |  |  |  |
| 22-ازالة الاشياء الصغيره من امام الطفل ومنعه من وضعها فى فمه من  الممكن ان يؤدى لمنع حدوث الاختناق والشرقه |  |  |  |
| 23- هل يجب اعطاء الطفل كميات قليلة من الطعام فى فمه ومناسبة لسنه؟ |  |  |  |
| 24- هل من الضرورى التاكد من مضغ الطفل الطعام جيدا قبل البلع؟ |  |  |  |
| 25- هل يجب عدم اضحاك الطفل اثناء الاكل؟ |  |  |  |

**ماهى مصادر معلوماتك عن الحوادث المنزليه؟ ( يسمح باكثر من اجابة):**

- وسائل الاعلام ( تلفزيون—انترنت- صحف....)
- الاقارب
- الطبيب
- حملات توعية
- اخرى ( حددى)

**المواقف السلوكية:**

**برجاء اختيار اجابة واحده لكل سؤال**

| **المواقف السلوكية** | **اوافق** | **أحيانا** | **لااوافق** |
| --- | --- | --- | --- |
| 1. هل تؤيدين إعطاء أساسيات الاسعافات الاولية وكيفية تفادى الحوادث كمنهج أساسي في المدارس والجامعات؟ |  |  |  |
| 2-هل تعتقدين أنه من الهام وجود حقيبة اسعافات اولية كاملة المحتويات في منزلك ؟ |  |  |  |
| 3 - هل تعتقدين ان التوعية بخصوص الحوادث المنزلية ستقلل من حدوثها ؟ |  |  |  |
| 4- ما هو تقيمك لهذه العبار" الحوادث المنزلية يمكن منعها" ؟ |  |  |  |
| 5-هل ترغبين فى حضور ندوات توعيه عن كيفيه التعامل وتفادى الحوادث المنزلية ؟ |  |  |  |
| 6- فى حالة حدوث اى من الحوداث المنزليه هل تعتقدين ان اول تصرف يجب القيام به هو طلب الخدمة الصحية فى اقرب مستشفى او وحدة صحية ؟ |  |  |  |
| 7- هل تعتقدين ان استخدام الاساليب التقليدية فى علاج الحوادث المنزليه قد يجدى نفعا فى علاجها ؟ |  |  |  |
| 8-عند حدوث اى من الحوداث المنزليه هل تعتقدين ان اول تصرف يجب القيام به هو استشارة الاقارب او الجيران ؟ |  |  |  |
| 9-هل تعتقدين انه من الهام مراقبة الام لطفلها طول الوقت وعدم تركه بمفرده فى المنزل ؟ |  |  |  |
| 10-هل تعتقدين انك قادرة على القيام بالاسعافات الاوليه لطفلك فى حال حدوث اى اصابة قبل الذهاب به للمستشفى ؟ |  |  |  |
| 11-هل تعتقدين انه من السهل حماية طفلك من التعرض للحوداث المنزليه ؟ |  |  |  |

**الفاعلية الذاتية:**

**برجاء اختيار اجابة واحده لكل سؤال**

| **الفعالية الذاتية** | **اوافق**  **بشدة** | **اوافق** | **احيانا** | | **لا اوافق** | **لا اوافق بشدة** | |
| --- | --- | --- | --- | --- | --- | --- | --- |
| 1- انا قادرة على تطبيق الاسعافات الاولية لطفلى في الحالات الطارئة قبل طلب الرعاية الصحية |  |  |  | |  |  | |
| 2- انا قادرة على القيام بتثقيف أسرتى على الإسعافات الأولية في حالات الإصابات المختلفة |  |  |  | |  |  | |
| 3-اذا قمت بالتخطيط لمنع حدوث الحوادث المنزليه فانا قادره على تطبيق ذلك |  |  |  | |  |  | |
| 4-اذا فشلت فى منع حدوث الحوادث المنزليه مره فلن استسلم وسأظل احاول حتى انجح فى ذلك |  |  |  | |  |  | |
| 5-اذا فشلت فى منع حدوث الحوادث المنزليه مره فلن احاول مرة اخرى |  |  |  |  | | |  |
| 6- ساقوم بالمبادرة فورا بتطبيق اجراءات السلامة بمنزلى |  |  |  |  | | |  |
| 7- تطبيق اجراءات منع الحوادث المنزليه صعب بالنسبة لى لذلك لا ستطيع القيام به |  |  |  |  | | |  |
| 8-الفشل يعنى بداية جديدة بالنسبة لى |  |  |  |  | | |  |
| 9-انا غير متاكده من قدرتى على القيام بحماية طفلى |  |  |  |  | | |  |
| 10- طبيعتى ان ايأس بسهوله |  |  |  |  | | |  |
| 11- انا غير مؤهلة ذاتيا للقيام باجراءات منع الحوادث المنزلية |  |  |  |  | | |  |
